# The Effect of Tele-Continuous Care on Maternal Functioning and Neonatal Perception among Iranian Primiparous Mothers: A Randomized Field Trial Study

**DOI:** 10.3390/jcm13206062

**Published:** 2024-10-11

**Authors:** Fatemeh Arang, Jennifer L. Barkin, Malihe Farid, Mahnaz Akbari Kamrani

**Affiliations:** 1 Student Research Committee, Medicine Faculty, Alborz University of Medical Sciences, Karaj, Iran; 2 Department of Community Medicine, Mercer University School of Medicine, Macon, GA 31207, USA; 3 Non-Communicable Diseases Research Center, Alborz University of Medical Sciences, Karaj, Iran; 4 Social Determinants of Health Research Center, Alborz University of Medical Sciences, Karaj, Iran

**Keywords:** midwifery, continuity of care, telehealth, maternal functioning, postpartum, perception

## Abstract

**Background/Objectives**: New mothers face significant challenges during the postpartum period, which can impact their maternal performance. This study aimed to assess the effect of tele-continuous midwifery care on maternal functioning and neonatal perception in first-time Iranian mothers. **Methods**: A randomized controlled field trial was conducted from January to May 2023 on 48 first-time mothers in the postpartum ward at Arash Women’s Hospital, Tehran. Participants were randomly assigned to either an intervention or control group. The intervention group received tele-continuous care for six weeks postpartum, while the control group received standard care. The Barkin Index of Maternal Functioning (BIMF) and the Neonatal Perception Inventory (NPI) were used to collect data in the second and sixth weeks after delivery. Data were analyzed using SPSS 26. **Results**: The mean age was 26.2 ± 4.8 years in the intervention group and 28.0 ± 6.1 years in the control group. An independent *t*-test revealed a significant difference in maternal functioning (BIMF score) between the intervention and control groups by the sixth week postpartum (*p* < 0.0001). A significant improvement in BIMF scores was observed within the intervention group from the second to the sixth week (*p* = 0.007). However, the McNemar’s test on the NPI showed no significant difference in the proportions of negative and positive maternal perceptions within the intervention group (*p* = 0.219) and in the control group (*p* = 0.508). **Conclusions**: Tele-continuous midwifery care effectively enhances maternal functioning during the vulnerable postpartum period, highlighting the necessity of ongoing support for new mothers.

## 1. Introduction

Motherhood is characterized as a transformative period during which a woman undergoes various multidimensional changes following pregnancy and childbirth, ultimately leading to the formation of her identity as a mother [1]. While many women report a sense of fulfillment in their maternal role, they simultaneously confront anxieties regarding the obligations that come with caring for an infant [2]. This is especially true for first-time mothers, who often find the experience of childbirth and the subsequent postpartum phase particularly overwhelming, as they must rapidly adapt to new responsibilities, such as breastfeeding, which can present significant physical and psychological challenges [3,4,5,6]. Insufficient knowledge, a deficiency in skills related to newborn care, and unpreparedness for future challenges contribute to heightened anxiety and significant exhaustion among new mothers. These factors can lead to sensations of time scarcity, a sense of losing control over personal circumstances, and increased levels of stress [7].

The concept of “Maternal Functioning” encompasses a range of maternal attributes by evaluating its multiple dimensions. According to this definition, a woman who obtains adequate social support maintains her physical and mental health, nurtures her infant, establishes a connection with her child, effectively manages her diverse responsibilities, and demonstrates adaptability over time is regarded as operating at an optimal level [8]. The substantial influence of enhanced maternal functioning on multiple dimensions of women’s lives and the quality of care provided to infants underscores the necessity for healthcare professionals and policymakers to advocate for positive childbirth experiences and to bolster mothers’ self-confidence [9]. Additionally, complications during the postpartum phase can impede the ideal development of the infant. Consequently, a thorough assessment of maternal functioning in the postpartum period may provide valuable insights into the mother’s ability to adapt effectively to her maternal responsibilities [10]. Additionally, mothers who hold favorable views of their newborns are more inclined to participate in activities that enhance the infant’s health, including breastfeeding and responsive caregiving. These practices have been linked to improved long-term outcomes for both mothers and their children [11]. Early intervention and support aimed at maternal mental health have been demonstrated to markedly enhance the quality of interactions between mothers and infants while also mitigating the negative impacts of postpartum depression on child development [12].

The postpartum phase, although critical, has historically received insufficient attention from both societal and healthcare perspectives. Recent health statistics indicate that a significant proportion of maternal mortality cases occur after childbirth, prompting increased scrutiny and emphasis on maternal health during this period from decision-makers and influential stakeholders, including researchers, policymakers, and commercial entities [13]. The World Health Organization (WHO) underscores the significance of thorough postpartum care as an essential aspect of maternal health. The objectives encompass the promotion of proper nutrition, the prevention and identification of illnesses, the encouragement of breastfeeding to guarantee sufficient nutrition for the infant, and the provision of reproductive health services [14,15]. The American College of Obstetrics and Gynecologists (ACOG) committee opinion emphasizes that to optimize the health outcomes for women and infants, postpartum care should be viewed as an ongoing process rather than a singular, isolated event. This care should be customized to align with the individual preferences of each woman, ensuring that services and support are appropriately tailored [16].

Research suggests that women often lack adequate professional support following their discharge from the hospital, which is essential for their adjustment to new roles [17]. Notwithstanding the establishment of postnatal care initiatives, complications and maternal mortality continue to be potential risks, even following childbirth [18]. In addition, obstacles such as the inadequate availability of mental health services, insufficient guidance for postpartum women regarding how to access care, and the absence of stable relationships between patients and healthcare professionals contribute significantly to the underutilization of postpartum care [19]. A significant number of women tend to favor a holistic approach that encompasses thorough, personalized, and constructive discussions regarding their specific needs during the postpartum phase [20].

The model of midwifery continuity of care is gaining traction in numerous developed nations, including New Zealand, Australia, England, Denmark, Canada, Switzerland, and Norway [21]. It is presented as a reliable, secure, and evidence-supported service for women [22]. The concept of continuity of care in midwifery includes relational, informational, and management aspects, ensuring that care is delivered consistently by the same midwife or a small team during pregnancy, labor, and the postpartum phase while also facilitating referrals to specialists when necessary. This model has demonstrated beneficial health outcomes for both mothers and infants, including decreased mortality rates and hospital admissions, enhanced patient satisfaction, and an overall positive birthing experience [23,24]. Continuous midwifery care has resulted in optimal satisfaction regarding prenatal, labor, and postpartum care, particularly for women who are at low risk of experiencing medical complications [25]. This care model facilitates the establishment of a relationship grounded in trust, equality, informed choice, decision-making, and shared responsibility between each woman and her midwife or a small team of midwives. Consequently, education, counseling, and care are tailored to meet the unique needs of each individual [26].

To maintain uninterrupted care, it is essential that mothers have access to healthcare services at all times, including during nights and holidays [27]. In contemporary healthcare, telehealth and digital health platforms have emerged as vital elements in the interactions between patients and clinicians. The shift to telemedicine, prompted by the COVID-19 pandemic, served as an unintentional experiment in healthcare accessibility across multiple medical disciplines. Postpartum appointments, which traditionally consisted of a single in-person visit that frequently experienced low attendance six weeks post-delivery, were abruptly transitioned to telehealth or video conferencing platforms [13]. The COVID-19 pandemic has led to an increase in feelings of isolation among many mothers, who have reported a notable decline in social support, especially in terms of emotional and practical assistance. This lack of support has intensified mental health challenges, including postpartum depression (PPD) and anxiety, which may hinder the development of a robust mother–infant relationship. Consequently, it is essential to evaluate and enhance social support systems to promote adequate adjustment and optimal functioning in mothers [28].

This study emphasizes the critical role of continuous midwifery care, supplemented by digital educational resources, messaging applications, and phone counseling as integral components of a comprehensive approach. This combined strategy aims to optimize postpartum support, enhancing maternal functioning during the crucial postpartum period. In light of the advancements in communication infrastructure in Iran, including the expansion of network coverage and the adoption of modern technologies (such as 4G, broadband, and fiber optics), alongside the increased utilization of telehealth services prompted by the COVID-19 pandemic, this study seeks to implement a model of midwifery continuity of care that incorporates telehealth. The objective is to deliver ongoing education, counseling, and care through messaging applications and educational technologies during the initial six weeks postpartum to the primiparous mothers. This research is designed to evaluate the impact of tele-continuous care on maternal functioning and perceptions of the newborn among Iranian primiparous mothers.

## 2. Materials and Methods

### 2.1. Study Design and Participants

This research constitutes a parallel, randomized, controlled field trial that included 48 first-time mothers who were admitted to the postpartum unit of Arash Women’s Hospital in Tehran, the capital city of Iran. These participants met the established inclusion criteria and demonstrated a willingness to engage in the study. The criteria for inclusion in the study required participants to be Iranian nationals, fluent in Persian, at least 18 years of age, married, and primiparous. Additionally, they needed to have delivered a singleton, healthy baby at term, possess a smartphone, and be capable of using messaging applications. The participants should not have any known medical or psychological conditions as self-reported, should not be addicted, and must not have a history of substance, psychotropic drugs, or alcohol use. Exclusion criteria included any serious medical issues arising in the mother or newborn during the study period. Due to the fact that baby blues generally manifest within the first two weeks after childbirth, the administration of the Edinburgh Postnatal Depression Scale (EPDS) was deferred until that period. A score of 15 or higher on the EPDS was established as the criterion for exit.

### 2.2. Ethics

This study was approved by the Ethics Committee of Alborz University of Medical Sciences (IR.ABZUMS.REC.1401.246). All the participants signed an informed consent letter and were allowed to leave the study at any stage.

### 2.3. Sample Size

The calculation of the sample size was performed utilizing the formula outlined below, specifically for a two-mean test concerning a quantitative trait of the sample, with a confidence level of 95% and a statistical power of 90%. This calculation was informed by the findings of the research conducted by Chamgurdani et al. (2020) [29], which reported the means and standard deviations of the total score of maternal functioning (M1 = 73.1, SD1 = 8.5) and (M2 = 95.8, SD2 = 11.8), alongside the parameters *µ*_1_ = 133.94, *µ*_2_ = 159.86, *σ*_1_ = 15.62, and *σ*_2_ = 17.83. Consequently, the sample size was established with 20 mothers for each group. To account for an expected dropout rate of approximately 20%, the sample size for both the intervention and control groups was adjusted to 24, culminating in a total sample size of 48 participants.

$$n=\frac{{\left(Z_{1}-\frac{\alpha }{2}+Z_{1}-\beta \right)}^{2}(\sigma_{1}^{2}+\sigma_{2}^{2})}{{\left({µ}_{1}-{µ}_{2}\right)}^{2}}$$

### 2.4. Allocation of Participants

**Figure 1:**
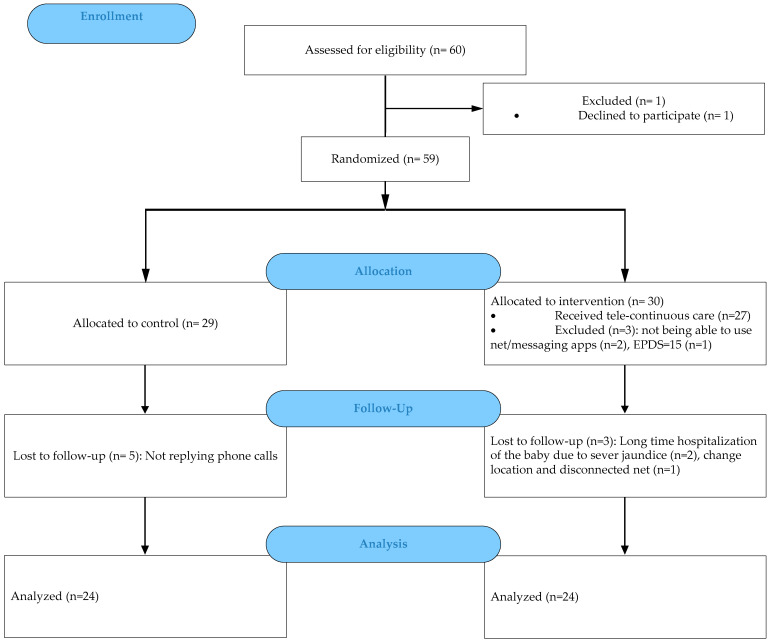
CONSORT flow diagram.

The samples were assigned to two intervention and control groups through the application of a random allocation rule. Since the list of all samples was not available at the beginning of the study, there was also a possibility that the proximity of the intervention and control group participants (in the postpartum ward) had an effect on the results of the study. It was decided that the sampling should be conducted for each group in different weeks. A lottery system was used to select the week. The CONSORT flow diagram for the study is as follows (Figure 1).

### 2.5. Data Collection Tool

The instruments employed in this research comprised a questionnaire assessing demographic and obstetric characteristics, the Barkin Index of Maternal Functioning (BIMF) to ascertain the maternal functioning scores, and the Neonatal Perception Inventory scale (NPI) to measure the maternal perceptions of the newborn.

#### 2.5.1. Demographic and Obstetric Questionnaire

The demographic and obstetric factors assessed comprised age, length of marriage, ethnicity, maternal education, paternal education, maternal occupation, paternal occupation, family income level, pregnancy intentions, delivery method, labor interventions, pregnancy complications, type of prenatal care received, and any history of miscarriage or stillbirth, as well as infertility. The final two inquiries regarding exclusive breastfeeding and postpartum visits to health centers were conducted six weeks following childbirth.

#### 2.5.2. Barkin Index of Maternal Functioning (BIMF)

The Barkin Index of Maternal Functioning (BIMF) is a self-administered questionnaire aimed at evaluating the status of maternal functioning. Created by Barkin et al., this tool is grounded in a holistic, patient-centered framework and demonstrates a Cronbach’s alpha of 0.87. It comprises 20 items distributed across seven domains: self-care, infant care, mother-infant interaction, social support, psychological wellness, management, and maternal adjustment. The respondents rate each item using a 7-point Likert scale, where 0 signifies strong disagreement, and 6 indicates strong agreement, resulting in total scores that range from 0 to 120. Elevated scores reflect enhanced maternal functioning, with a maximum score of 120 denoting optimal functioning [10]. The psychometric properties of the BIMF have been substantiated within an Iranian demographic, achieving reliability and validity with a Cronbach’s alpha of 0.88 and an intra-class correlation coefficient (ICC) of 0.85 [30]. Furthermore, the Persian adaptation of the Barkin questionnaire was validated by Ansariniaki et al. in 2021, yielding a reliability coefficient of 0.77, an ICC of 0.87, and a Cronbach’s alpha of 0.81 [31]. In the current investigation, the reliability of the questionnaire was reaffirmed, showing a Cronbach’s alpha of 0.726 in the second week and 0.761 in the sixth week postpartum.

#### 2.5.3. Neonatal Perception Inventory Scale (NPI)

The Neonatal Perception Inventory (NPI) scale, created by Broussard in 1971, serves to evaluate maternal perceptions regarding their newborns. This scale is divided into two sections, each comprising six questions that pertain to specific behavioral traits of the newborn: crying, feeding, fecal elimination, restlessness, sleeping, and spitting up. In the initial section, mothers provide responses based on their general impressions of a typical newborn, while in the subsequent section, they assess their individual newborn. Mothers are instructed to compare their newborns to the average newborn they envision, responding to the questions using a 5-point Likert scale. The disparity in scores between the two sections reflects the mother’s perception of her child; a score of zero or lower indicates a negative perception, whereas a score exceeding zero signifies a positive perception [32]. The validity of the Persian adaptation was assessed by Ghafouri et al., with contributions from ten faculty members at the Iran University School of Nursing, utilizing a 5-point Likert scale (1 = never, 2 = very little, 3 = moderate amount, 4 = a good bit, and 5 = a great deal). Reliability was established with a Cronbach’s alpha of 0.76 [33]. In the current study, the reliability of the questionnaire was reaffirmed, yielding a Cronbach’s alpha of 0.691 during the second week and 0.721 by the sixth week of the investigation.

### 2.6. Procedure

Following the selection of eligible samples within the department, effective communication was established, and a sociodemographic questionnaire was completed. Basic health education was then provided in person by the midwife, covering topics such as episiotomy care, breastfeeding, recognizing danger signs, managing postpartum pain, and umbilical cord care. Subsequently, the most suitable messaging platform for ongoing communication and questionnaire distribution was identified to implement tele-continuous midwifery care after discharge from the hospital. For the participants in the intervention group, a subscription to health TV educational media was acquired from https://www.behdasht.tv (accessed on 21 January 2023), granting access to authentic educational videos in Farsi. Over the six weeks following delivery, individuals in the intervention group received continuous midwifery care and education, including full-time support to address their questions and individual concerns, as well as counseling via phone calls, text messages, messaging applications, and access to educational videos. Moreover, the same puerperium health tips were sent daily to all mothers of the intervention group during these six weeks, covering the following key topics: maternal and neonatal danger signs, postpartum visit schedule, breastfeeding, infant care, postpartum screening tests, postpartum care, postpartum exercise, and paternal role. In contrast, the control group only received standard postpartum care after hospital discharge. The standard postpartum care in Iran, according to the Ministry of Health protocols, includes multiple in-person visits for the mother and newborn. These visits involve newborn screenings for thyroid and metabolic diseases and follow-up examinations. Receiving this care requires both the mother and newborn to visit health centers on designated days. Upon completion and submission of the sixth-week questionnaires, those interested were offered free midwifery consultations via phone or messenger, addressing their health inquiries and counseling needs. Participants from both the control and intervention groups were instructed to fill out the questionnaires during the second and sixth weeks following delivery and to submit a photograph of their responses via the designated messaging platform. For those individuals who were unable to submit their responses punctually, the researcher conducted phone interviews to facilitate the completion of the questionnaires.

### 2.7. Data Analysis

The obtained data were analyzed using SPSS V26 software. Descriptive statistics, including the mean, standard deviation, and absolute frequency, were utilized. The Kolmogorov–Smirnov test was used to determine whether the data distribution was normal. For analytical statistics, independent *t*-tests, chi-square, and the Exact Fischer’s test, or their non-parametric equivalents (Mann–Whitney and Wilcoxon tests) were used.

## 3. Results

In the present study, 24 participants were involved in the data analysis of each intervention and control group. The mean ages of the participants in the intervention and control groups were 26.208 ± 4.809 and 27.958 ± 6.139 years, respectively. Two groups were homogeneous in terms of the demographic variables (Table 1) and obstetric history (Table 2).

Independent *t*-tests on the BIMF results showed that the total mean scores did not differ significantly between the intervention and control groups in the second week (*p*-value = 0.1). However, the total mean scores of the BIMF differed significantly between the two groups, and it was higher in the intervention group six weeks after receiving tele-continuous care compared to the control group (*p*-value = 0.0001) (Table 3).

Additionally, the paired sample *t*-test on the BIMF results showed that the mean difference in the total scores between the second and sixth weeks was statistically different in the intervention group (*p*-value = 0.007). Thus, the total mean score increased by 7.333 in the intervention group. In contrast, no significant difference was observed in the BIMF scores between the second and sixth weeks on the mean difference in the total scores in the control group (*p*-value = 0.282) (Table 4).

According to the findings, there was no statistically significant difference between the various domains of maternal functioning in the second week between the intervention and control groups. Whereas, by the sixth week, there were statistically significant differences in the mean scores of most domains of maternal performance, such as mother–infant interaction (*p*-value = 0.022), social support (*p*-value = 0.025), psychological well-being (*p*-value = 0.0001), and management (*p*-value = 0.0001) between the intervention and control groups (Table 5). These differences were due to the increase in the mean scores of self-care, mother–infant interaction, maternal mental health, and management in the intervention group from the second week to the sixth week.

Regarding the NPI scale, the paired sample *t*-test results indicated no significant difference in the mean scores of maternal perceptions of (typical/individual) newborn behavior between the second and sixth weeks in the intervention (*p*-value = 0.238) and control groups (*p*-value = 0.758). Similarly, no significant difference was found in the mean scores of maternal perceptions of their own newborn’s behavior between the second and sixth weeks in the intervention (*p*-value = 0.151) and control groups (*p*-value = 0.496) (Table 6).

The application of McNemar’s test on the NPI results indicated that there is no statistically significant difference in the proportions of negative and positive maternal perceptions in the second and sixth weeks following continuous care across the two groups (Table 7).

## 4. Discussion

This study investigated the impact of tele-continuous midwifery care on maternal functioning and neonatal perception among first-time mothers. The results revealed a significant difference in the total BIMF scores between the intervention and control groups, with the intervention group demonstrating notably higher scores six weeks post-receipt of tele-continuous midwifery care. These findings underscore the effectiveness of such care in enhancing the functioning of primiparous women, suggesting that participants in the intervention group exhibited a greater capacity to adjust to the postpartum phase. Furthermore, these results align with the favorable outcomes reported in prior research concerning midwifery continuity of care [25,34,35,36]. Recent studies suggest that ensuring continuity of care in midwifery may serve as a preventive strategy to reduce maternal anxiety, worries, and depressive symptoms during the perinatal period [35]. This underscores the importance for healthcare providers to consider both the physical and psychosocial dimensions of care throughout pregnancy and the postpartum phase [37].

Research was carried out by Susanti et al. (2021), focusing on the continuity of midwifery care in Indonesia and the initiation and evolution of a mobile health application (mHealth). The qualitative phase of the study, which involved focus group discussions with 13 midwives, revealed that midwives providing continuity of care require the mHealth application to enhance their learning and the delivery of health services, including counseling and education via telemidwifery. These functionalities facilitate the early identification and management of pregnancy complications, thereby aiding in the prevention of issues during childbirth, as well as for newborns and in the postpartum phase. Additionally, midwives find the application advantageous for the streamlined recording and reporting of maternal and child health (MCH) data, as it allows them to conveniently access these features from their smartphones at any time and place. Nonetheless, it is important to highlight that midwives in developing countries often possess limited skills in utilizing mobile health applications for the provision of continuous care [38]. Studies suggest that the criteria for maternal healthcare in Iran require a comprehensive assessment to guarantee the provision of high-quality, safe, and woman-centered maternal care for mothers and their families [39]. Furthermore, the development of immediate messaging solutions is enhancing communication capabilities, which can complement postpartum home visits and promote woman-centered care. Nonetheless, it is imperative that authorities and relevant organizations ensure equitable access to these services for all women. [40].

The design of our study was centered on delivering midwifery care, counseling, and educational support to first-time mothers, commencing in the postpartum ward immediately following delivery and extending through the sixth week post-childbirth. Throughout this timeframe, the personal concerns and inquiries of the mothers were addressed via messaging applications or telephone communications. Additionally, educational videos were incorporated as a resource to assist mothers during their postpartum recovery. The findings indicated a notable enhancement in the psychological well-being, mother–infant interaction, management skills, and social support aspects of the mothers’ functioning within the intervention group by the conclusion of the sixth week. A comparable investigation was carried out in Iran by Chamgurdani et al. (2019) in Tabriz, which sought to examine the effects of skill-based counseling on maternal functioning among postpartum women. The study involved four group counseling sessions, each lasting between 60 to 90 min, with a one-week interval between sessions, commencing from the fourth week postpartum. The post-intervention BIMF questionnaires were administered two weeks following the final session. The findings revealed that the total BIMF score for the intervention group was significantly elevated two weeks after the skill-based counseling sessions when contrasted with the control group (*p* < 0.001). Furthermore, notable improvements were recorded across all domains of maternal functioning post-intervention [29]. These outcomes align with the results of the current study.

The findings from the NPI results of this study indicate that there were no notable changes in the intervention group between the second and sixth weeks postpartum. This suggests that tele-continuous midwifery care may exert a stabilizing influence on mothers’ perceptions of their newborns.

Additionally, a study by Aba and Komurcu in Turkey in 2016, which focused on the effects of prenatal education on adaptation during pregnancy, postpartum adjustment, and adolescent mothers’ perceptions of their newborns (ages 15–19), revealed no significant differences in the NPI results between the experimental and control groups (*p* = 0.627) [32]. This finding aligns with the results of the current study. Nevertheless, the design of the previous study, which concentrated solely on prenatal education for adolescent mothers, does not completely correspond with the outcomes of the present research, which emphasized the provision of tele-continuous postnatal care for mothers aged 18 and older.

Hesse et al. (2021) conducted a qualitative study grounded in psychoanalytic theory, highlighting the importance of addressing mothers’ mental health alongside their physical well-being during childbirth and the subsequent stages of motherhood. The study posits that akin to the advantages infants gain from a supportive emotional environment, mothers equally necessitate a caring and nurturing setting throughout pregnancy and delivery. Consequently, it is imperative for systems and cultural practices surrounding childbirth to place greater emphasis on emotional dimensions to enhance the support provided to mothers [41].

A descriptive investigation by Simsek et al. (2022) explored the perceptions and adaptive behaviors of mothers concerning their infants. The findings indicate that mothers experience a sense of comfort and reassurance when provided with information regarding newborn nutrition, sleep patterns, and both early and late care practices, as well as when their inquiries are addressed [2]. Consistent with these results, first-time mothers, who often face stress and uncertainty, greatly appreciated the ongoing consultations and training during the postpartum period through various means such as phone calls, messaging applications, and educational videos.

The limitations of this study warrant attention, particularly the dependence on self-reported questionnaire completion, which presupposed the accuracy of the participants’ answers. Furthermore, the elevated stress levels experienced by primiparous mothers, stemming from physical, emotional, and relational challenges during the crucial postpartum phase, may have influenced their responses. Consequently, it is advisable for future research to conduct similar studies with a larger sample size and an extended follow-up period, ideally spanning 4 to 6 months postpartum, while employing team-based methodologies.

## 5. Conclusions

These findings emphasize the importance of ongoing support and education for new mothers beyond the prenatal period to enhance postpartum functioning. Tele-continuous care, as demonstrated in the present study, can play a crucial role in improving postpartum adaptation and maternal functioning in primiparous women. The notable increase in maternal functioning, particularly in domains directly related to maternal–infant interaction and mental health, highlights the importance of tailored postpartum support programs. Also, the findings underscore the importance of integrating telehealth interventions into postpartum care strategies to support maternal functioning and infant care practices. Therefore, initiation, progression, and execution of tele-continuous midwifery care, particularly during the critical postpartum period, are recommended. Further research is recommended to explore the long-term benefits of such interventions and to identify the most effective strategies for supporting new mothers during the critical postpartum period.

## Figures and Tables

**Table 1:** Sociodemographic Characteristics of study participants by groups.

Variable	Tele-Continuous Care Group (*n* = 24)	Control Group (*n* = 24)	*p*
**Age (Mean ± SD)**	26.208 ± 4.804 *	27.958 ± 6.139 *	0.277 ^a^
**Marriage Duration (Mean ± SD)**	3.34 ± 2.07 *	2.88 ± 1.53 *	0.234 ^b^
**Ethnicity**	***n* (%)**	***n* (%)**	0.61 ^c^
Fars	11 (45.8)	6 (25)	
Turk	3 (12.5)	8 (33.3)	
Lor	8 (33.3)	5 (20.8)	
Kurd	0 (0)	4 (16.7)	
Other	2 (8.3)	1 (4.2)	
**Mother’s Education**			0.978 ^c^
Elementary	2 (8.3)	2 (8.3)	
First High School	3 (12.5)	3 (12.5)	
Second High School	3 (12.5)	5 (20.8)	
Diploma	10 (41.7)	9 (37.5)	
University	6 (25)	5 (20.8)	
**Husband’s Education**			0.956 ^c^
Elementary	2 (8.3)	2 (8.3)	
First High School	3 (12.5)	4 (16.7)	
Second High School	2 (8.3)	3 (12.5)	
Diploma	11 (45.8)	11 (45.8)	
University	6 (25)	4 (16.7)	
**Mother’s job**			1 ^c^
Housewife	22 (91.7)	23(95.8)	
Employee	2 (8.3)	1(4.2)	
**Husband’s job**			0.140 ^c^
Worker	5 (20.8)	9 (37.5)	
Employee	3 (12.5)	0 (0)	
Freelancer	16 (66.7)	14 (58.3)	
Other	0 (0)	1 (4.2)	
**Family income level**			0.083 ^c^
Weak	15 (62.5)	9 (37.5)	
Medium	9 (37.5)	15 (62.5)	
Good	0 (0)	0 (0)	

* The numbers show means (standard deviations); ^a^ *p*-values of independent *t*-test; ^b^ *p*-values of Mann–Whitney test; ^c^ *p*-values of Chi-square/Exact Fischer’s test.

**Table 2:** Obstetrics characteristics of study participants by groups.

Variable	Tele-Continuous Care Group (*n* = 24)	Control Group (*n* = 24)	*p*
**Pregnancy Intention**	***n* (%)**	***n* (%)**	0.234 ^c^
Wanted	24 (100)	21 (87.5)	
Unwanted	0 (0)	3 (12.5)	
**Delivery Type**			0.221 ^c^
Vaginal delivery	10 (41.7)	6 (25)	
Cesarian section	14 (58.3)	18 (75)	
**Intervention during Labor**			1 ^c^
Yes	0 (0)	0 (0)	
No	24 (100)	24 (100)	
**Pregnancy Complications (** **such as gestational diabetes, high blood pressure)**			0.137 ^c^
Yes	2 (8.3)	7 (29.2)	
No	22 (91.7)	17 (70.8)	
**Pregnancy Care**			0.247 ^c^
Office	15 (62.5)	12 (50)	
Health Center	6 (25)	11 (45.8)	
Both	3 (12.5)	1 (4.2)	
**History of Abortion/Stillbirth**			0.303 ^c^
Yes	7 (29.2)	4 (16.7)	
No	17 (70.8)	20 (83.3)	
**History of Infertility**			0.701 ^c^
Yes	5 (20.8)	3 (12.5)	
No	19 (79.2)	21 (87.5)	
**Exclusive Breastfeed**			0.562 ^c^
Yes	12 (50)	14 (58.3)	
No	12 (50)	10 (41.7)	
**Postpartum visit to Health centers**			0.551 ^c^
Yes	14 (58.3)	16 (66.7)	
No	10 (41.7)	8 (33.3)	

^c^ *p*-values of Chi-square/Exact Fischer’s Test.

**Table 3:** Total mean scores of the BIMF at the second and sixth weeks after continuous care.

Variable and Group		Minimum	Maximum	Mean	SD	*p*
2nd week	Intervention	57.00	108.00	91.041	12.167	0.1
	Control	66.00	106.00	85.541	10.442	
6th week	Intervention	73.00	114.00	98.375	11.860	0.0001
	Control	76.00	103.00	87.083	7.546	

**Table 4:** Mean difference in BIMF score (week 2 to week 6).

Group	Mean Difference (Week 2 to Week 6)	SD	*p*
Intervention	−7.333	12.038	0.007
Control	−1.541	6.858	0.282

**Table 5:** Comparison of mean scores of the BIMF domains between groups at the second and sixth weeks after continuous care.

	Intervention Group (*n* = 24) Mean (SD) *	Control Group (*n* = 24) Mean (SD) *	*p*
**Self-Care**			
2nd week	12.333 (3.318)	12.291 (3.972)	*p* = 0.969 ^a^
6th week	13.750 (2.658)	12.291 (3.406)	*p* = 0.119 z = −0.557 ^b^
**Infant care**			
2nd week	11.125 (1.034)	11.000 (1.215)	*p* = 0.282 z = −1.075 ^b^
6th week	10.708 (1.366)	10.666 (1.372)	*p* = 0.322 z = −0.991 ^b^
**Mother–infant interaction**			
2nd week	12.750 (3.096)	14.666 (2.334)	*p* = 0.093 z= −1.678 ^b^
6th week	11.250 (3.504)	12.791 (2.812)	*p* = 0.022 z= −2.290 ^b^
**Psychological well-being**			
2nd week	43.875 (6.250)	41.708 (5.908)	*p* = 0.223 ^a^
6th week	48.791 (6.135)	42.208 (4.232)	*p* = 0.0001 z = −3.574 ^b^
**Social support**			
2nd week	13.333 (4.124)	11.750 (3.416)	*p* = 0.154 ^a^
6th week	13.541 (4.772)	11.500 (3.706)	*p* = 0.025 z = −2.237 ^b^
**Management**			
2nd week	26.000 (5.133)	23.291 (4.582)	*p* = 0.060 ^a^
6th week	27.958 (4.515)	23.166 (4.114)	*p* = 0.0001 ^a^
**Adjustment**			
2nd week	9.833 (1.992)	10.041 (1.428)	*p* = 0.858 z= −0.179 ^b^
6th week	10.541 (1.768)	10.166 (1.372)	*p* = 0.175 z = −1.355 ^b^

* Mean (standard deviation); ^a^ *p*-values of independent *t*-test; ^b^ *p*-values of Mann–Whitney U.

**Table 6:** Total mean scores of the NPI scores at the second and sixth weeks after continuous care.

Group and Variables			NPI Scores				*p*
			Min	Max	Mean	SD	
**Intervention**	General impressions of a typical newborn	Week 2	14.00	23.00	17.083	2.602	0.238
		Week 6	10.00	22.00	16.500	2.670	
	General impressions of their individual newborn	Week 2	9.00	19.00	14.416	2.430	0.151
		Week 6	9.00	21.00	15.041	2.804	
**Control**	General impressions of a typical newborn	Week 2	14.00	24.00	17.7083	2.45798	0.758
		Week 6	12.00	25.00	17.5417	2.85869	
	General impressions of their individual newborn	Week 2	9.00	21.00	15.1250	3.91555	0.496
		Week 6	9.00	21.00	15.5833	3.67029	

*p*-values of the paired sample *t*-test.

**Table 7:** Status of the mother’s perception status at the second and sixth weeks after continuous care.

Group		Mother’s Perception’s Status		χ^2^-Statistic (df) ^a^	*p*-Value
		Negative (6th Week) *n* (%)	Positive (6th Week) *n* (%)		
**Intervention**	Negative (2nd week)	5	1	5.714	0.219
	Positive (2nd week)	5	13		
**Control**	Negative (2nd week)	5	3	1.343	0.508
	Positive (2nd week)	6	10		

^a^ McNemar’s Chi-squared test with continuity correction.

## Data Availability

The data are available if requested.
